# Ising Model for Interpolation of Spatial Data on Regular Grids

**DOI:** 10.3390/e23101270

**Published:** 2021-09-28

**Authors:** Milan Žukovič, Dionissios T. Hristopulos

**Affiliations:** 1 Department of Theoretical Physics and Astrophysics, Faculty of Science, Pavol Jozef Šafárik University in Košice, Park Angelinum 9, 04154 Košice, Slovakia; 2 School of Electrical & Computer Engineering, Technical University of Crete, 73100 Chania, Greece; dchristopoulos@ece.tuc.gr

**Keywords:** Ising model, spatial classification, interpolation, non-Gaussian data, earth observation, fast algorithm, 02.50.-r, 02.50.Ga, 02.60.Ed, 75.10.Hk, 89.20.-a, 89.60.-k

## Abstract

We apply the Ising model with nearest-neighbor correlations (INNC) in the problem of interpolation of spatially correlated data on regular grids. The correlations are captured by short-range interactions between “Ising spins”. The INNC algorithm can be used with label data (classification) as well as discrete and continuous real-valued data (regression). In the regression problem, INNC approximates continuous variables by means of a user-specified number of classes. INNC predicts the class identity at unmeasured points by using the Monte Carlo simulation conditioned on the observed data (partial sample). The algorithm locally respects the sample values and globally aims to minimize the deviation between an energy measure of the partial sample and that of the entire grid. INNC is non-parametric and, thus, is suitable for non-Gaussian data. The method is found to be very competitive with respect to interpolation accuracy and computational efficiency compared to some standard methods. Thus, this method provides a useful tool for filling gaps in gridded data such as satellite images.

## 1. Introduction

The current availability of massive remotely sensed georeferenced datasets, pertaining to land cover, terrain elevation, population, meteorological variables, and atmospheric pollution creates increasing demands for efficient processing and analysis methods. The information contained in such Earth-observation data can help to develop reliable tools for ecosystem management, environmental policy, the design of real-time hazard warning systems, and various other decision-making tasks. However, the Earth-observation data typically require preprocessing before they can be used in standard analytic methods. A typical problem is data heterogeneity, i.e., the fact that data are acquired by different modalities, using different methodologies and space-time resolutions. Furthermore, data coverage is often incomplete due to different reasons, such as limited resources (material, human, and technical), equipment limitations (detection level and resolution), sensor malfunctions, or adverse meteorological conditions (observations hindered by clouds) [1,2].

Resolution differences between different sensors as well as data gaps create the missing data problem. In order to apply standard tools for the analysis of space-time earth-observation data, there is a need to fill the gaps and to unify the resolution. These tasks involve downscaling (refining) data with sparse resolution and generating optimal estimates at points without measurements. The mathematical problem of gap filling is interpolation: Estimates of the variable under consideration need to be generated at the target point based on the available data in the vicinity of the target point. Depending on the nature of the modeled variable, interpolation involves either a classification problem (if the values of the variable come from a set of class labels) or regression (if the variable is continuous or if its values are classes that correspond to closed intervals of real numbers). The interpolation in such cases can be performed by means of well established interpolation and classification techniques [3,4] or by means of machine learning methods [5].

However, considering the ever increasing size of spatial data, both classical methods used in geostatistics (e.g., kriging and its various flavors) and machine learning methods (e.g., Gaussian processes) can become impractical due to their high computational complexity [4,5,6]. Namely, the complexity of such methods increases proportionally to the third power of the data size which renders their application impossible without modification (see [4,5] and references therein for possible alternatives). Furthermore, methods such as kriging usually assume a joint Gaussian probability distribution, an assumption that is often unjustified by the data. In addition, application of such methods typically requires considerable human (subjective) input [7,8].

An alternative approach focuses on modelling spatial correlations by means of short-range interactions, inspired from models of statistical physics [9,10,11]. These works focused on the development of computationally efficient spatial dependence models applicable to gridded and scattered Gaussian data. Spatial data on regular grids are often modeled by means of Gaussian Markov random fields [12] with spatial correlations imposed via local interactions, which allow for computationally efficient representations. However, there is considerably less progress on the development of non-Gaussian Markov random fields [13]. The prototypical non-Gaussian Markov random field is the binary-valued Ising model, which has found applications mostly within the spin-glass theory, applied to the image restoration problem [14,15,16,17,18]. In spatial statistics, the Ising model was introduced in the works of Besag [19]. The Ising model was introduced for data with binary values. Nonetheless, it can also be applied to multi-valued discrete data within a hierarchical framework that employs multiple binarization thresholds [20,21].

The objective of the current study is to investigate the performance of the Ising nearest neighbor correlation (INNC) interpolation method (originally introduced in [20,21]) with real datasets of environmental interest (soil quality and terrain elevation data), as well as synthetic data. The analysis of the latter allows us a controlled assessment of the prediction ability and computational performance of the algorithm for different data sizes. One of the specific goals of this study is to establish the potential of INNC for interpolating massive (e.g., satellite product) datasets. Another goal is to evaluate INNC’s ability to handle different probability distributions, a property which derives from the method’s non-parametric nature. Finally, INNC is investigated with respect to its ability to generate accurate predictions with minimal user input, a property that makes it an appealing candidate for the automatic gap-filling of massive datasets.

## 2. Definition of the Interpolation Problem

Let us consider a set of sampling points $G_{s}=\left\{{\vec{r}}_{i}\right\}$, where ${\vec{r}}_{i}=\left(x_{i},y_{i}\right)\in R^{2}$ and i=1,…,N. These points are assumed to be distributed on a rectangular grid $\tilde{G}$ of size $N_{G}=L_{x}\times L_{y}$, where $L_{x}$ and $L_{y}$ are, respectively, the horizontal and vertical dimensions of the rectangle (in terms of the unit length) and $N<N_{G}$. Let $z_{i}$ be a value attributed to the point ${\vec{r}}_{i}$. Then, the set $Z_{s}={\left\{z_{i}\in R\right\}}_{i=1}^{N}$ represents the sample of the process. Let $G_{p}={\left\{{\vec{r}}_{p}\right\}}_{p=1}^{P}$ be the set of prediction points where p=1,…,P such that $\tilde{G}=G_{s}\cup G_{p}$.

For label data, $Z_{s}$ takes values in a set of discrete labels ${\left\{C_{q}\right\}}_{q=1}^{N_{c}}$. For regression applications, $Z_{s}$ can be represented as a realization of an underlying continuously valued random field $Z({\vec{r}}_{i})$. In the following, we discretize the continuous distribution using a number of classes, $C_{q},\;q=1,\ldots ,N_{c}$. The classes are defined with respect to a set of threshold levels $t_{k},\;k=1,\ldots ,N_{c}+1$, where $t_{1}=\min\left(z_{1},\ldots ,z_{N}\right)$ and $t_{N_{c}+1}=\max\left(z_{1},\ldots ,z_{N}\right).$ Each class $C_{q}$ corresponds to an interval as follows: $C_{q}=\left(t_{q},t_{q+1}\right]$ for $q=2,\ldots ,N_{c}-1$, $C_{1}=\left(t_{1},t_{2}\right]$, and $C_{N_{c}}=\left(t_{N_{c}-1},t_{N_{c}}\right).$

We define the *indicator field* $I_{Z}\left({\vec{r}}_{p}\right)$ to take integer values $q\in \{1,\ldots ,N_{c}\}$ equal to the appropriate class index for the value $z({\vec{r}}_{p})$. In particular, $I_{Z}\left({\vec{r}}_{p}\right)=q$ implies that $\hat{z}\left({\vec{r}}_{p}\right)\in C_{q}$ (where $C_{q}$ is a specific label for the classification problem or a specific interval for the regression problem). The interpolation problem can then be posed as a classification problem for both classification and regression applications, i.e., each point in $G_{p}$ is assigned a class label. In order to estimate the class identity of *Z* at the prediction points, we use the well-studied Ising model. Once all the prediction points have been assigned to a class, a map of the process *Z* can be generated consisting of equivalent class (isolevel) contours.

## 3. The Ising Model

For each class *q*, let us assume a set of variables ${\left\{s_{i}^{q}\right\}}_{i=1}^{N}$ (“spins”) that can take the value $s_{i}^{q}=1$ (“spin-up”) or $s_{i}^{q}=-1$ (“spin-down”). The Ising model considers pairwise interactions between the spins, expressed by the following Hamiltonian (for brevity, we drop the class index) [22]:

(1) $$\begin{array}{c} H\left[\left\{s\right\}\right]=-\sum_{i,j}J_{i,j}\;s_{i}\;s_{j}-\sum_{i}h_{i}s_{i}, \end{array}$$

where the symbol H[{s}] denotes that energy is a function of the set of spin values (spin configuration).

In general, spin configurations that result in lower energy are more likely to be realized. The first term in the energy corresponds to the “spin–spin exchange" interaction energy. The coupling strength $J_{i,j}$ controls the strength as well as the type of the interaction: if $J_{i,j}>0$, it is “ferromagnetic” (it favors spins of the same sign), but if $J_{i,j}<0$ it is “antiferromagnetic” (favoring spins of the opposite sign). The second term corresponds to a symmetry-breaking bias, which is caused by the presence of a site dependent “external field” $h_{i}$. Positive (negative) values of the external field favor spins of the same sign. Hence, $h_{i}$ controls the overall distribution of the “spin” values between 1 and −1 (the magnetization). The coupling strength $J_{i,j}$ is usually considered to be uniform, and its range limited to nearest neighbors. However, the model can be generalized to include also non-uniform, longer-range couplings.

The probability density function for a spin configuration {s} is given by the following Boltzmann–Gibbs exponential expression:

(2) $$\begin{array}{c} f\left[\left\{s\right\}\right]=\frac{e^{-H\left[\left\{s\right\}\right]/k_{B}T}}{Z}, \end{array}$$

where $k_{B}$ is Boltzmann’s constant and *T* the temperature. The partition function *Z* is a normalization factor obtained by summing the exponential $e^{-H\left[\left\{s\right\}\right]/k_{B}T}$ over all possible “spin configurations”. Hence, it is only a function of the model parameters $J_{i,j}$ and $h_{i}$ but not of a particular configuration.

In the forward problem, the coupling strength and the polarizing field are known, and one is interested in the most probable spin configurations or in the calculation of the spin correlation function. In the inverse problem, the “spins” at certain locations are known (they can be obtained from the sampled field values). The estimation process focuses on inferring the model parameters (e.g., by means of the maximum likelihood method) that best represent the observations. Unfortunately, the normalizing constant *Z* is in most cases intractable by analytical means, and its numerical evaluation is a computational bottleneck. Possible approaches to circumvent this problem, such as the maximum pseudo-likelihood approach [23] or various Markov Chain Monte Carlo estimation techniques [24], can be either very inaccurate or prohibitively slow, respectively.

Once the model parameters are determined, the optimal values of the “spins” at non-sampled locations (i.e., where the data gaps are), can be determined by maximizing the conditional (on the data) probability *f* (equivalently by minimizing *H*) with respect to the unknown values.

In order to circumvent the difficult problem of parameter estimation, we use a non-parametric method, explained below, that does not require knowledge of the Ising model’s parameters.

## 4. The INNC Multilevel Interpolation Algorithm

### 4.1. Non-Parametric Nearest-Neighbor Model

In the following, we use the ideas motivating the Ising Hamiltonian (Equation (1)). In this study, we restrict the scope of the Ising model to the simplest energy functional: We set the polarizing field uniformly to zero, i.e., $h_{i}=0,i=1,\ldots ,N$ and limit the exchange interactions to uniform “ferromagnetic” strength only for nearest neighbors (N.NB.), i.e., $J_{i,j}=J>0$ if i∈N.NB.(j) and $J_{i,j}=0$ otherwise. The choice of zero polarizing field does not allow explicitly controlling the ratio of “up” versus “down spins”. As explained below, this is achieved in the simulations by selecting the initial “spin” values so as to reflect the “up-down” spin distribution of the sample.

If we are dealing with an interpolation problem that involves only two classes, the interpolation is performed in a single pass. The “data” $Z_{s}={\left\{z_{i}\right\}}_{i=1}^{N}$ are transformed into discrete variables (“spins”). If our problem involves multiple classes (resulting from different labels or from the discretization of continuous value), a hierarchical interpolation scheme is used. In this scheme, the sample, $G_{s}^{q}$, and prediction, $G_{p}^{q}$, sub-grids are progressively updated as the class index *q* changes from 1 to $N_{c}$. For the lowest class $G_{s}^{1}=G_{s}$ and $G_{p}^{1}=G_{p},$ where $G_{s}$ and $G_{p}$ are the initially defined sampling and interpolation grids, respectively. For all classes, $q=1,\ldots ,N_{c}$, $G_{p}^{q}\cup G_{s}^{q}=\tilde{G}$.

As we increase *q*, the sites with negative spins join the updated sample subgrid, and they are simultaneously removed from the prediction subgrid. At each level *q*, the discretization is binary with respect to the respective threshold value, i.e., $s_{i}^{q}=-1$ if $z_{i}\leq t_{q+1}$ and $s_{i}^{q}=1$ if $z_{i}>t_{q+1}$ for $i=1,\ldots ,N_{q}$, where $N_{q}$ is the number of sites with known values at level *q*. For q>1, the sample (prediction) subgrid is augmented (diminished) by the grid nodes ${\vec{r}}_{l}\in G_{p}$ for which $s_{l}^{q-1}=-1.$ It follows that $N_{1}=N$ and $N_{q>1}\geq N$. The set $S_{s}^{q}={\left\{s_{i}^{q}\right\}}_{i=1}^{N_{q}}$ where $q=1,\ldots ,N_{c}$ includes all the spin values for the class index *q*. The union of the two sets containing the sample and prediction values at level *q*, i.e., ${\tilde{S}}^{q}=S_{s}^{q}\cup S_{p}^{q}$ contains the “spin” values over the entire grid $\tilde{G}$ for the specific level. The Ising model can then be used to represent spatial interactions between the spins ${\tilde{S}}^{q}$ for level *q*, which means that the spins are defined with respect to the corresponding binarization threshold.

The hierarchical scheme outlined above helps to avoid the parameter inference problem and suggests a non-parametric approach. This approach utilizes a cost function, $U(S_{p}^{q}|S_{s}^{q}),$ that measures the deviation (squared difference) between a suitably normalized energy, $C_{s}^{q}$, of the sample configuration at level *q* and the respective energy of the spin configuration ${\tilde{C}}^{q}$ over the entire grid. This is given by the following:

(3) $$\begin{array}{c} U\left(S_{p}^{q}|S_{s}^{q}\right)={\left({\tilde{C}}^{q}-C_{s}^{q}\right)}^{2}, \end{array}$$

where $C_{s}^{q}={\langle s_{i}^{q}\;s_{j}^{q}\rangle }_{G_{s}^{q}}$ is the spin pair correlation of the sample configuration at the q−level, and ${\tilde{C}}^{q}={\langle s_{i}^{q}\;s_{j}^{q}\rangle }_{\tilde{G}}$ is the spin pair correlation over the entire grid; the latter includes both $S_{p}^{q}$ and $S_{s}^{q}$.

Thus, assigning the correct class to the spins $S_{p}^{q}$ is reduced to finding the optimal configuration ${\hat{S}}_{p}^{q}$, which minimizes the cost function (3) at a fixed temperature *T*.

(4) $$\begin{array}{c} {\hat{S}}_{p}^{q}=\underset{S_{p}^{q}}{arg min}U\left(S_{p}^{q}|S_{s}^{q}\right). \end{array}$$

### 4.2. Hierarchical Strategy

The hierarchical algorithm proceeds sequentially at the lowest binarization threshold and proceeds by increasing the class index. The binary discretization and the classification of the non-measured sites are initially performed with respect to the first class and then repeated sequentially for the remaining classes. The “gaps” in the prediction subgrid, $G_{p}$, are gradually filled as the algorithm proceeds through consecutive levels. At each level, all the locations identified as having −1 spin values at the lower levels are used as input (sample data) in the current stage. The reduced prediction subgrid, $G_{p}^{q}$, for the class index *q* contains $P^{q}$ points so that for $q>q^{\prime }$ it holds that $P^{q}\leq P^{q^{\prime }}$ and $P^{1}=P$. In the case of continuous variables, the classes $C_{q}$ can be defined as desired and do not need to represent intervals of uniform size.

The INNC algorithm uses the *rejection ratio*, which is defined by the following.

$$\rho =\frac{\mathrm{number}\;\mathrm{of}\;\mathrm{rejected}\;\mathrm{states}}{\mathrm{number}\;\mathrm{of}\;\mathrm{simulated}\;\mathrm{states}}.$$

The rejection ratio is constantly updated and is used to control when the algorithm should stop proposing new states and move on to the next class level *q*.

The main steps of the INNC method are shown in the pseudocode of Algorithm 1.

This algorithm returns an indicator field ${\hat{I}}_{Z}=I_{Z}\left(G_{s}\right)\cup {\hat{I}}_{Z}\left(G_{p}\right)$, which consists of the original sample classes and the class estimates at $G_{p}$. The indicator values at the sampling sites are exactly reproduced because the initial state respects these values and the iterative steps skip over sites in the updated sample set $S_{s}^{q}$. Below, we refer to $I_{Z}\left(G_{s}\right)$ as the *training set*.

Note that Algorithm 1 is presented for non-vectorized implementation, but the generation of new states (line 12) is actually realized using vectorized single-spin Metropolis updating. The vectorization is enabled owing to the fact that the square grid can be divided into two interpenetrating subgrids in a checkerboard fashion (checkerboard decomposition). Hence, by considering the short-range character of the interaction restricted to the nearest neighbors, the spins in the first subgrid only interact with spins of the second subgrid and vice versa. By means of this decomposition, it is possible to apply the updating algorithm to spins belonging to the same subgrid in parallel. The algorithm sweeps through the lattice several times until the rejection ratio exceeds the threshold value (herein, it is set to one).

Spin updating is performed at zero temperature. The T=0 constraint means that there is no stochastic selection of unfavorable spins. Hence, candidate “spins” to be updated are flipped unconditionally only if the flip lowers the cost function. This is called a “greedy” Monte Carlo algorithm [25], and it guarantees convergence, which is usually very fast. In comparison, in simulated annealing, *T* is slowly lowered starting from an initial high-temperature state. This approach is much slower computationally, but the resulting configuration is less sensitive to the initial state ${\hat{S}}_{p}^{q\;(0)}$. The sensitivity of the greedy algorithm is known to be especially pronounced in high-dimensional spaces with non-convex energies. In such cases, the greedy algorithm is more likely to become stuck in the local minima instead of converging to the global minimum. However, this is not a concern for the interpolation problem. In fact, targeting the global minimum of the cost function *U* strongly emphasizes the sample correlation energy per “spin” pair $C_{s}^{q}$, ignoring that the latter is influenced by sample-to-sample fluctuations.
**Algorithm 1:** Summary of INNC interpolation algorithm in the non-vectorized version. The vectorized version is explained in the text.
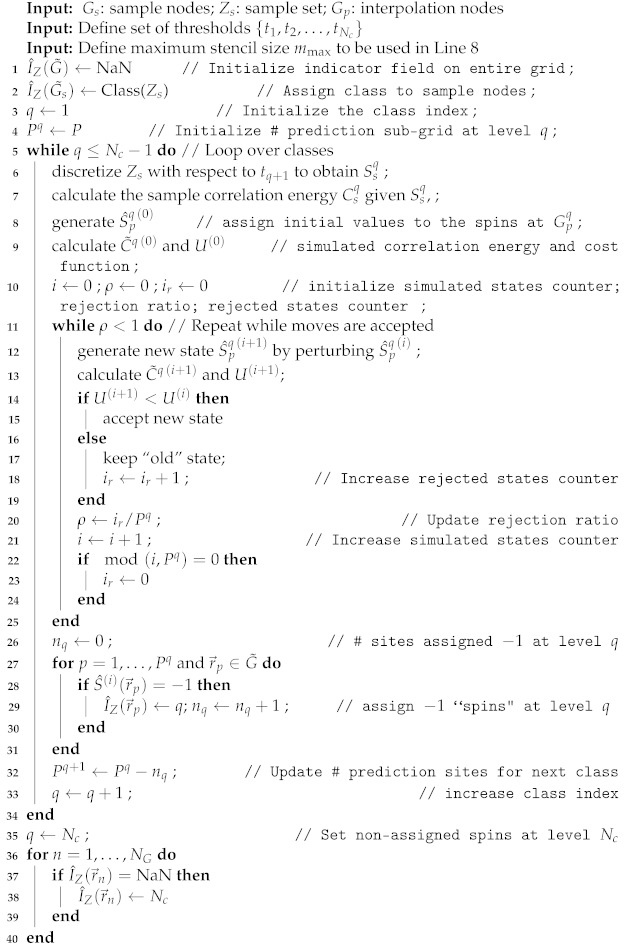



The initial configuration can be selected with a number of methods. Since the proposed model aims to provide a fast and automatic interpolation method, the initial configuration should minimize the relaxation path (in state space) to the equilibrium. It should also be selected preferably with little or no user intervention. Assuming a certain degree of spatial continuity, which is common in geospatial data sets, ${\hat{S}}_{p}^{q\;(0)}$ is determined based on the “augmented sample” states $S_{s}^{q}$ in the immediate neighborhood of each individual prediction point. The neighborhood of such a node ${\vec{r}}_{p}$ is determined by an adaptable m×m stencil (where m=2l+1) centered at ${\vec{r}}_{p}$. The stencil size $m\leq m_{\max}$ is adaptively determined, reflecting local sampling density and spin value distributions. Starting from an initial value of m=3, we tested if a clear majority of either positive or negative spin values is established within the stencil. If this is not the case, we increased *m* by one, tested again, and repeated testing as necessary. An arbitrary upper bound $m_{\max}$ is imposed on the stencil size to prevent oversmoothing and to restrict the computational load (memory and CPU time). Then, ${\hat{s}}_{p}^{q\;(0)}$ is assigned by majority rule, based on the prevailing value of its neighbors in $S_{s}^{q}$ inside the stencil. If there is no prevailing sign (i.e., if an equal number of +1 and −1 values are present or if ${\vec{r}}_{p}$ has no neighbors in $S_{s}^{q}$ inside the stencil), the initial value is randomly assigned.

The proposed INNC updates are accepted unconditionally as long as they lower the cost function of Equation (3). Using the vectorized checkerboard algorithm, the entire grid is swept in two steps. The simulation terminates for a given class index *q* if one complete sweep through the interpolation subgrid $G_{p}^{q}$ does not produce a single successful update. The hierarchical scheme used implies that the computational load is reduced with increasing *q*, which is in line with the reduction in size of the subgrid $G_{p}^{q}$.

The input information required by the algorithm, thus, involves the definition of the class intervals and the maximum stencil size $m_{\max}$ used to generate the initial state. The number of classes depends on the nature of the problem and the objective of the study: If the interpolation problem involves discrete class labels, the number of labels is predetermined and no discretization is needed. If the interpolation problem involves a continuous-valued process, the discretization depends on the objective of the study. If the goal is to determine exceedance levels, binary classification is sufficient. For environmental monitoring and decision-making purposes, a moderate number (e.g., six or eight) of classes is often sufficient. For example, the Fire Weather Index used to measure fire risk in Europe is mapped into six classes (very low, low, medium, high, very high, and extreme) [26]. However, a higher number of classes can be used if one desires to resolve the values of the modeled process in a superior manner.

## 5. Data Description

The performance of the INNC interpolation method is demonstrated with two environmental data sets as well as with synthetic (simulated) data. The first set represents a map of soil quality data categorized in different classes. This data set contains a finite number of discrete levels; thus, the prediction of missing data is inherently a classification problem.

The second data set represents a map of surface elevation; the latter is a continuously valued variable. For the purpose of generating an isolevel elevation map corresponding to some predefined resolution, the elevation data should be discretized according to the desired resolution to allow applying INNC.

The above two environmental data sets are used to assess the classification and regression performance of INNC for gap filling. Both data sets exhibit a skewed non-Gaussian probability distribution, which allows testing the ability of INNC to operate under non-Gaussian conditions.

Finally, we generate synthetic sets of spatially correlated data of different sizes. These enable us to assess the computational complexity and ability of INNC to automatically fill gaps in very large data sets, such as remote sensing images.

### 5.1. Soil Quality

This data set describes soil quality for crop production over a major part of Europe and is obtained from the Harmonized World Soil Database. The latter is a 30 arc second raster database with over 16,000 different soil mapping units that combines existing regional and national updates of soil information worldwide with the information contained within the 1:5,000,000 scale FAO-UNESCO Soil Map of the World [27].

Our chosen data represent the soil nutrient availability segregated in seven classes according to the degree of constraints imposed on soil quality (1—no or slight; 2—moderate; 3—severe; 4—very severe; 5—mainly no soil; 6—permafrost; 7—water). In this classification, lower numbers correspond to “better” soil quality. The spatial domain considered is a rectangle of 120×85 pixels. Some summary statistics are as follows: size $N_{G}=$ 10,200, $z_{\min}=1$, $z_{\max}=7$, $\bar{z}=1.785$, $z_{0.50}=2$, and $\sigma_{z}=0.984$. The value of the skewness coefficient is 2.02 and of the kurtosis coefficient 9.632. The frequency histogram of soil quality class values is presented in Section 7.1.

### 5.2. Surface Elevation

This data set represents the surface elevation on a 5 min latitude/longitude grid over part of the territory of North America (approximately 80°–110° W, 55°–40° N). The data form a rectangle comprising 400×200 pixels [28]. Elevation values (in meters) are referenced to the center of each cell with a resolution of 1 m.

Some summary statistics are as follows: size $N_{G}$ = 80,000, $z_{\min}=1$ m, $z_{\max}=3790$ m, $\bar{z}=774.41$ m, $z_{0.50}=441$ m, and $\sigma_{z}=713.17$ m. The skewness coefficient is equal to 1.37, and the kurtosis coefficient is equal to 4.07. As evidenced from the above statistics, the data are non-Gaussian and positively skewed. The elevation frequency histograms corresponding to the respective class intervals considered in the study are presented in Section 7.2, Section 7.3 and Section 7.4.

### 5.3. Synthetic Data

The synthetic data are simulated from the joint Gaussian distribution with mean m=50 and standard deviation equal to σ=10, i.e., Z∼N(m=50,σ=10). The spatial correlations are imposed by means of the exponential covariance $C\left(r\right)=\sigma^{2}\exp\left(-r/\xi \right)$, where ξ=5 and *r* represents the Euclidean distance between any two grid nodes. The exponential covariance function implies that the spatial process is relatively rough and, thus, is appropriate for modeling, e.g., soil processes.

The data are generated on square grids with *L* nodes per side, where L=32,…,2048 using the spectral simulation method [4,29,30]. The largest grid size examined is typical of data sets collected by various remote sensing techniques.

## 6. Missing Data Simulation and INNC Performance Assessment

In order to generate data sets with missing data (gaps), we follow the methodology described below. From each complete data set, we generated a partial sample $Z_{s}$ of size $N=\left(1-p\right)N_{G}$ by randomly removing $P=pN_{G}$ nodes. The removed values are set aside for validation purposes. For three different degrees of thinning, p=0.33,0.5, and 0.66, we generate 100 different partial sample configurations. These differ from each other with respect to the set of grid nodes that have been removed. The values of the process at these validation nodes are then estimated by using the INNC interpolation Algorithm 1.

In order to assess INNC performance, the estimated values at the validation nodes are compared with the true values (which were removed from the respective sample). In classification performance evaluation, the indicator values $I_{Z}\left(G_{p}\right)$ at the validation nodes are compared with the estimates ${\hat{I}}_{Z}\left(G_{p}\right)$, obtained after removing the set of nodes $G_{p}$ from the data. The originally discrete data (soil quality) are used without further processing.

To test the interpolation performance of continuously valued data (surface elevation and synthetic), we first discretize the data according to the desired resolution. We use different resolutions and respective class intervals. For the surface elevation data set, a resolution of 500 m is used first, which segregates the data into $N_{c}=8$ classes and $C_{q}=\left[500\left(q-1\right),\;500q\right)$, q=1,…,8. Second, a finer resolution of 250 m is used, resulting in $N_{c}=15$ classes corresponding to the intervals $C_{q}=\left[250\left(q-1\right),\;250q\right)$, q=1,…,15. Finally, we gradually increase the resolution up to $N_{c}=100$ classes in order to test the interpolation performance for data with almost continuous variations.

In the case of synthetic data, we arbitrarily discretize the entire range of observed values into $N_{c}=8$ classes and test the interpolation performance with increasing domain size. This design aims to study the scaling of INNC computational complexity with size. The INNC algorithm is applied in all cases with a maximum stencil size $m_{\max}=5$.

The measure that we use to assess interpolation performance of the INNC algorithm in the case of classification is the misclassification rate, i.e., the fraction of misclassified pixels defined by the following:

(5) $$\begin{array}{c} F=\frac{1}{P}\sum_{p=1}^{P}\left[1-\delta \left(I_{Z}\left({\vec{r}}_{p}\right),{\hat{I}}_{Z}\left({\vec{r}}_{p}\right)\right)\right], \end{array}$$

where *P* is the number of validation points, $I_{Z}\left({\vec{r}}_{p}\right)$ is the true value at the validation points, and ${\hat{I}}_{Z}\left({\vec{r}}_{p}\right)$ is the INNC estimate; $\delta (I,I^{\prime })=1$ if $I=I^{\prime }$, $\delta (I,I^{\prime })=0$ if $I\neq I^{\prime }$ is the Kronecker delta.

In the case of a large number of classes, the root mean square error (RMSE) is a more suitable measure for evaluating INNC interpolation performance. As will be shown in the following section, the RMSE typically shows a decrease with increasing $N_{c}$ up to some threshold $N_{c}^{*}$ beyond which it stabilizes and becomes independent of $N_{c}$. The value of $N_{c}^{*}$ depends on the data set under consideration, but it appears to decrease with sample sparseness. The RMSE is defined as the following:

(6) $$\begin{array}{c} RMSE=\sqrt{\sum_{p=1}^{P}\frac{1}{P}{\left[Z\left({\vec{r}}_{p}\right)-\hat{Z}\left({\vec{r}}_{p}\right)\right]}^{2}}, \end{array}$$

where $Z({\vec{r}}_{p})$ is the original true value at the point ${\vec{r}}_{p}$, and $\hat{Z}\left({\vec{r}}_{p}\right)$ is the estimate of the continuously valued field. This estimate is obtained from the classification of ${\hat{I}}_{Z}\left({\vec{r}}_{p}\right)$ and a subsequent back-transformation of the indicator field scale to the original continuum scale.

$$\hat{Z}\left({\vec{r}}_{p}\right)=t_{{\hat{I}}_{Z}\left({\vec{r}}_{p}\right)}+\frac{1}{2}\left(t_{{\hat{I}}_{Z}\left({\vec{r}}_{p}\right)+1}-t_{{\hat{I}}_{Z}\left({\vec{r}}_{p}\right)}\right).$$

In Section 7, we use RMSE to assess the interpolation of surface elevation data.

Average values of the misclassification rate and the RMSE, respectively, are obtained from ensembles of different missing-data realizations with the same degree of thinning.

The computations are performed in the Matlab® programming environment on a desktop computer with 16 GB of RAM and an Intel®Core ™i7-4790 processor with an 3.60 GHz clock.

## 7. Results

### 7.1. Soil Quality

**Figure 1:**
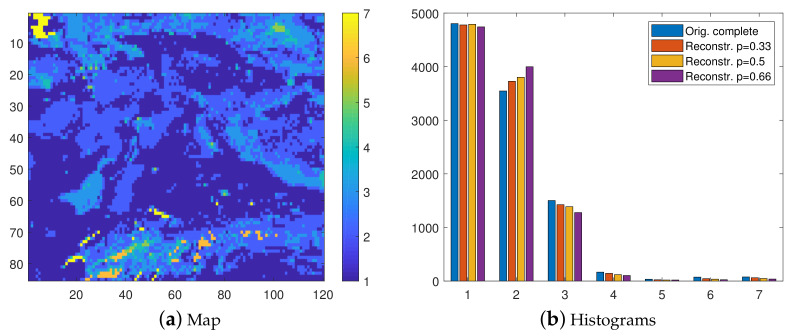
(**a**) Map of complete soil quality data. (**b**) Group of histograms. From left to right: Complete data and reconstructions from the thinned data with p=0.33,0.5 and 0.66, respectively. The histograms of the reconstructed data represent average values obtained from 100 realizations.

The map and the histogram of the complete data are shown in Figure 1. It is evident in these plots that the first three (1–3) classes clearly dominate over the remaining ones, covering almost 97% of the spatial domain. Figure 1b, in addition to the histogram of the class values for the complete data (left bar) also includes the histograms of the reconstructions by means of INNC classification for the three degrees of thinning (p= 0.33, 0.5, and 0.66). These histograms are shown by the respective bars 2–4 (moving from left to right) in Figure 1b.

The histograms (bars 2–4 in Figure 1b display mean values obtained from 100 realizations (missing data configurations for a given *p*). The match between the distributions of the original and the reconstructed data deteriorates with increasing *p*. In particular, the second class is overestimated at the cost of mainly the third class. Note that the second class is the closest to the mean value (calculated as the sum of the class values, each multiplied with the respective probability). Hence, the overestimation of the second class can be attributed to the averaging effect, which is common in interpolation methods.

**Figure 2:**
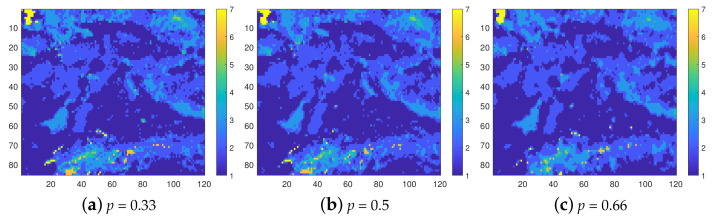
Class maps of the soil quality data, reconstructed from samples with thinning degrees p=0.33,0.5, and 0.66, respectively.

Figure 2 presents the reconstructed maps based on INNC. These maps are obtained from a single realization (the first from the ensemble of one hundred). As already suggested by the histograms in Figure 1b, the visual agreement between the original map (shown in Figure 1a) and the reconstructions deteriorates with increasing sparsity of the sampling subgrid. For example, for p=0.66, the most apparent misclassification is observed in the sixth class (permafrost, shown in orange color). This class appears in the complete data in very small and disconnected clusters, which are surrounded by bigger clusters that belong in different classes. Therefore, permafrost clusters can be viewed as “hot-spots”, which are difficult to predict particularly because sampling points in this class are sparse. Misclassification also occurs along the borders between different classes. Note that the values on the sampling subgrid $G_{s}$ (which varies between realizations) do not contribute to misclassification since INNC by construction honors the values on $G_{s}$.

Table 1 lists quantitative measures of the INNC classification performance based on statistics calculated over the ensemble of 100 realizations. These include the mean misclassification rate $\langle F^{*}\rangle$, the standard deviation ${\mathrm{STD}}_{F^{*}}$ of the misclassification rate, and CPU time $\langle T_{cpu}\rangle$. The above are complemented by measures intrinsic to the current method, such as the mean number of Monte Carlo steps, $\langle N_{MC}\rangle$, required to optimize the cost function and the mean cost function $\langle U^{*}\rangle$ at termination. The averaging of $N_{MC}$ and $T_{cpu}$ is performed over individual realizations for the cumulative values all the class levels. The averaging of the cost function $U^{*}$ is performed over both the ensemble of realizations and all class levels of each realization.

To validate the classification ability and computational performance of INNC, we compare it with the commonly used the fuzzy *k*-nearest neighbor (FKNN) classification algorithm [31] implemented in the Matlab^®^, function fknn [32]. We chose the FKNN method because it has been shown to dominate its non-fuzzy counterpart in terms of lower error rates and also to compare well with other standard more sophisticated classification methods. At the same time, it is still relatively simple and computationally efficient enough to process larger data sets (it would be computationally impossible to perform the analysis presented below by using some more sophisticated classification methods, such as Support Vector Machines). As expected, the misclassification rate increases with *p* for both methods. However, INNC exhibits superior performance. First, the INNC misclassification rate is lower than the FKNN method’s misclassification rate, i.e., $\langle F^{*}\left(INNC\right)\rangle /\langle F^{*}\left(FKNN\right)\rangle =0.54,0.56$, and 0.58 for p=0.33,0.50, and 0.66, respectively. At the same time, the computational speed of INNC exceeds that of FKNN by two orders of magnitude for all degrees of thinning, namely $\langle T_{cpu}\left(INNC\right)\rangle /\langle T_{cpu}\left(FKNN\right)\rangle =0.0128,0.0109$ and 0.0125 for p=0.33,0.50, and 0.66, respectively.

As for the remaining INNC measures, one can notice a slight increase in $\langle N_{MC}\rangle$ with *p*. However, the total number of steps remains very low, and the difference between the smallest value for p=0.33 and the largest value for p=0.66 does not exceed one MC sweep. There is also some increase in $\langle U^{*}\rangle$ with *p*, but all the values obtained reflect a satisfactory level of convergence to the optimum. We note that even though the greedy algorithm does not pursue global minima, the values of the cost function are quite close to zero.

### 7.2. Surface Elevation: Resolution 500 m—8 Classes

**Figure 3:**
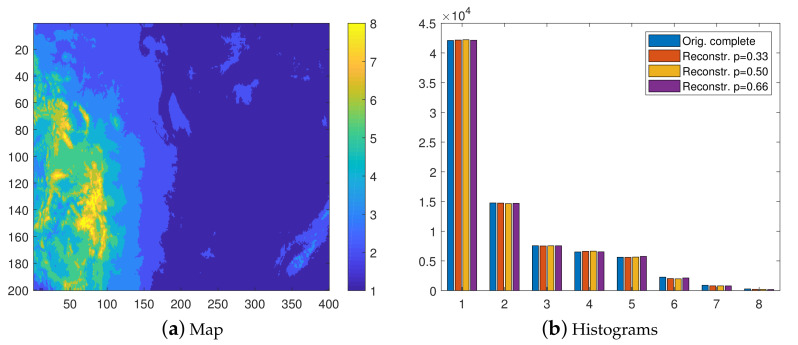
(**a**) Eight-class isolevel map of the complete surface elevation data at 500 m resolution. (**b**) Group of histograms. From left to right: complete data and the reconstructions based on the thinned data with p=0.33,0.5 and 0.66, respectively. The histograms of the reconstructions are based on mean values obtained from 100 realizations.

The isolevel map and the histogram of the complete data, discretized according to the vector of thresholds corresponding to this resolution are shown in Figure 3. In the map, the elevations in the range of 0≤Z<500 m (first class) dominate, covering about 55% of the area, while those above 3500 m correspond only to 0.1%. Figure 3b presents the histogram of the class values for the complete data (left histogram bar) as well as the histograms of the INNC reconstructions for the three degrees of thinning. The histograms of the reconstructions based on the training sets with p=0.33,0.5, and 0.66 are shown by the 2–4 (moving from left to right). The histograms represent average values obtained from 100 realizations. The match between the probability distributions of the complete data and the reconstructions is excellent. As mentioned above, this was achieved without explicit control by means of an external field, i.e., by using $h_{i}=0$ in Equation (1).

**Figure 4:**
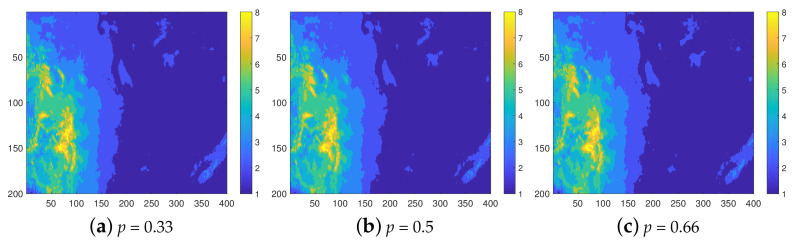
Class maps of elevation data in the 8-level classification scheme. The reconstructions are based on samples with thinning degrees p=0.33,0.5, and 0.66, respectively.

Figure 4 helps to visualize the interpolation results in terms of reconstructed maps. The isolevel maps are obtained from a single realization (the first from the set of one hundred). We observe that the reconstructed maps provide a close visual match to the original map, shown in Figure 3a. This is the case not only at lower *p* but also the spatial patterns of the original map are reconstructed surprisingly well at p=0.66.

The first part of Table 2 presents the quantitative measures of INNC interpolation performance along with the measures obtained by means of FKNN. As expected, the misclassification rates increase with *p* for both INNC and FKNN. However, the INNC misclassification rates are again much lower than those of FKNN method, i.e., $\langle F^{*}\left(INNC\right)\rangle /\langle F^{*}\left(FKNN\right)\rangle =0.74,0.75$ and 0.78 for p=0.33,0.50 and 0.66, respectively. However, in comparison with the soil quality data, the relative differences between the methods are smaller. On the other hand, the computational efficiency of INNC with respect to FKNN is even higher, i.e., $\langle T_{cpu}\left(INNC\right)\rangle /\langle T_{cpu}\left(FKNN\right)\rangle =0.004,0.004$, and 0.005 for p=0.33,0.50, and 0.66, respectively.

The values of $\langle N_{MC}\rangle$ are slightly higher than for the soil quality data, and their increase with *p* is more apparent. While the overall increase in $\langle N_{MC}\rangle$ for the surface elevation data can be ascribed to the increased size of the data set, the increase in $\langle N_{MC}\rangle$ with *p* can be generally ascribed to the fact that $\langle N_{MC}\rangle$ is a measure of the “spin” system’s relaxation time.

On the other hand, increasing *p* translates into higher *P* and, thus, a larger state-space of size $2^{P}$. Since the number of prediction nodes, $P^{q}$, decreases with *q* due to the progressive filling of gaps by the INNC hierarchical scheme, the Metropolis sampler tends to speed up as *q* increases. The relaxation time is shortened by proper choice of the initial state.

There are interlevel differences in the value of $U(S_{p}^{q}|S_{s}^{q})$, but their magnitudes remain relatively small. For example, even at p=0.66, which results in the highest values of the cost function, $\max\left(U^{*}\right)\leq 10^{-3}$. The average CPU time needed for the optimization at any *p* is of the order of a fraction of second. The very low values of $\langle N_{MC}\rangle$ and $\langle T_{cpu}\rangle$ are also due to the vectorized implementation of spin updating using the checkerboard algorithm.

### 7.3. Surface Elevation: Resolution 250 m—15 Classes

**Figure 5:**
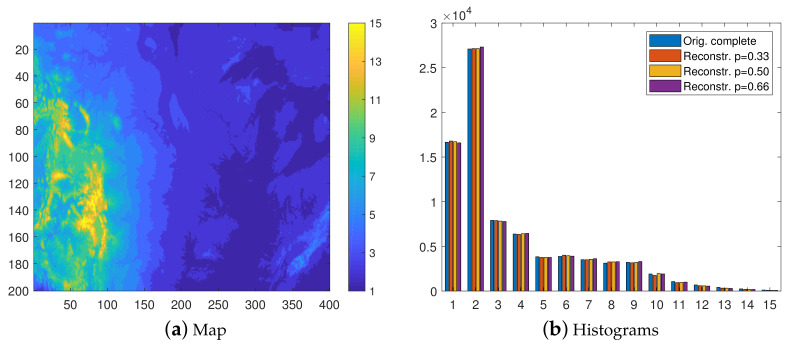
(**a**) Fifteen-class isolevel map of the complete data at 250 m resolution. (**b**) Group of histograms. From left to right: complete data and the reconstructions based on the thinned data with p=0.33,0.5, and 0.66, respectively. The histograms of the reconstructions are based on mean values obtained from 100 realizations.

Next, we repeat the classification experiment by using a resolution of 250 m. The isolevel map in Figure 5a clearly has higher resolution than the eight level map in Figure 3a. The most and least represented classes are the second and last, which contain approximately 34% and 0.1% of the values, respectively.

**Figure 6:**
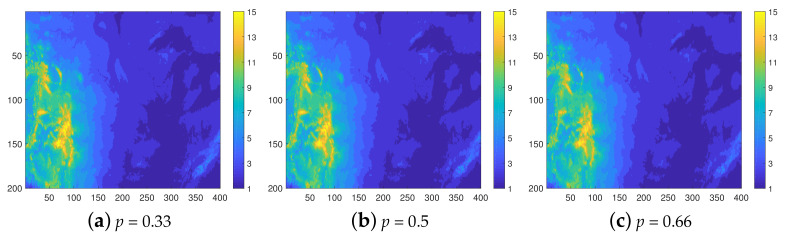
Class maps of elevation data in the 15-level classification scheme. The reconstructions are based on samples with thinning degrees p=0.33,0.5, and 0.66, respectively.

Due to the higher resolution, an increase in the misclassification rate is expected. Nevertheless, as is evident in Figure 5b and Figure 6, both the class distributions and the visual patterns are recovered quite well by the reconstructions in all cases.

By comparing the numerical values of $\langle F^{*}\rangle$ obtained by increasing the number of classes from 8 to 15, the misclassication rates almost doubles (see the second part of Table 2). Nevertheless, the ratio of the misclassification rates obtained by means of the INNC and FKNN methods, i.e., $\langle F^{*}\left(INNC\right)\rangle /\langle F^{*}\left(FKNN\right)\rangle =0.76,0.78$, and 0.81 for p=0.33,0.50, and 0.66, respectively, remains similar to the respective ratios for eight levels, showing only a slight increase.

On the other hand, INNC seems to exhibit a linear increase in computational time and $\langle N_{MC}\rangle$ with $N_{c}$; hence, these measures almost doubled when $N_{c}$ changed from $N_{c}=8$ to $N_{c}=15$. In contrast, the FKNN computational time increased only marginally. Therefore, the relative computational efficiency of the INNC method decreased, resulting in $\langle T_{cpu}\left(INNC\right)\rangle /\langle T_{cpu}\left(FKNN\right)\rangle =0.008,0.008$, and 0.010 for p=0.33,0.50, and 0.66, respectively. Hence, INNC remained quite competitive in terms of computational time with respect to FKNN.

### 7.4. Surface Elevation: Increasing Resolution—Crossover to Continuous Interpolation

It is interesting to investigate the interpolation performance of the INNC method for gradually increasing number of levels. However, for sufficiently large values of $N_{c}$, it makes more sense to evaluate prediction performance in terms of prediction errors, such as the RMSE defined in Equation (6). Then, INNC can be compared with a standard interpolation method. For this purpose we used the inverse distance weighted (IDW) method [33] implemented in the Matlab®  function fillnans [34]. The parameters for IDW were as follows: power = 2.7 and unlimited search radius.

**Figure 7:**
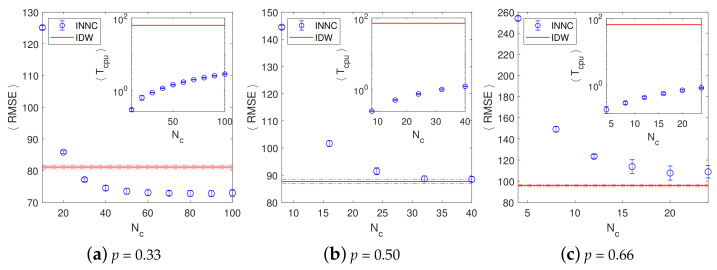
(**a**) 〈RMSE〉 and $\langle T_{cpu}\rangle$ as functions of the number of classes in the INNC and IDW methods. The mean values and error bars are obtained from 5–20 realizations.

In Figure 7 we present the evolution of the RMSE of INNC (blue circles) with increasing number of levels and compared it with the RMSE of IDW (red line) for different degrees of thinning *p*. In all the cases, one can observe a gradual decrease in RMSE with increasing $N_{c}$ up to a certain threshold value $N_{c}^{*}$, beyond which the RMSE levels off. This threshold point appears to decrease with increasing *p*: It corresponds to $N_{c}^{*}\approx 50$ for p=0.33, $N_{c}^{*}\approx 30$ for p=0.50, and $N_{c}^{*}\approx 20$ for p=0.66. In comparison to the IDW method, for p=0.33, the RMSE of INNC reaches the IDW value of 81.13±0.60 at $N_{c}\approx 25$ and beyond $N_{c}^{*}\approx 50$ it levels off at the value 72.88±0.92, where the errors represent one standard deviation.

The range of RMSE estimates is based on the ensemble of 100 realizations. However, for larger *p*, the relative superiority of the INNC method seems to diminish. For p=0.50, the optimal RMSE of the INNC method 88.55±1.03 achieved for $N_{c}>30$ is comparable with the IDW value of 87.68±0.81; for p=0.66, the IDW method is clearly superior with an RMSE 95.86±1.26 versus the optimal INNC value of 108.29±6.26 beyond $N_{c}^{*}\approx 20$. In addition, we observed that, for all values of *p*, the dispersion of the RMSE obtained by INNC is greater than that for IDW. Both of these patterns can be attributed to the fact that IDW uses information from the entire sample at each prediction node. This results in improved estimates compared to INNC, especially for sparser data sets (higher *p*). The improved performance of IDW compared to INNC for higher *p* is also due to the spatial patterns of the elevation, which exhibits spatial correlations that extend over a large portion of the grid. A different data set with less spatial continuity would be more favorable for INNC.

The insets in the respective panels of Figure 7 represent the computational efficiency of the two methods. They show the evolution of the CPU time of the INNC method (blue circles) with increasing $N_{c}$ and compared it with that of the IDW method (red line). Since the CPU time of INNC is relatively very small and only increases linearly with the number of levels (note the semi-log scale), one can conclude that even for $N_{c}≳N_{c}^{*}$, the INNC CPU times are on average about two orders of magnitude smaller than the CPU time of the IDW method. This behavior reflects the fact that INNC is a local method, while IDW takes into account all the data on the sample subgrid for the interpolation at each prediction node.

### 7.5. Synthetic Data: Scaling with Data Size

**Figure 8:**
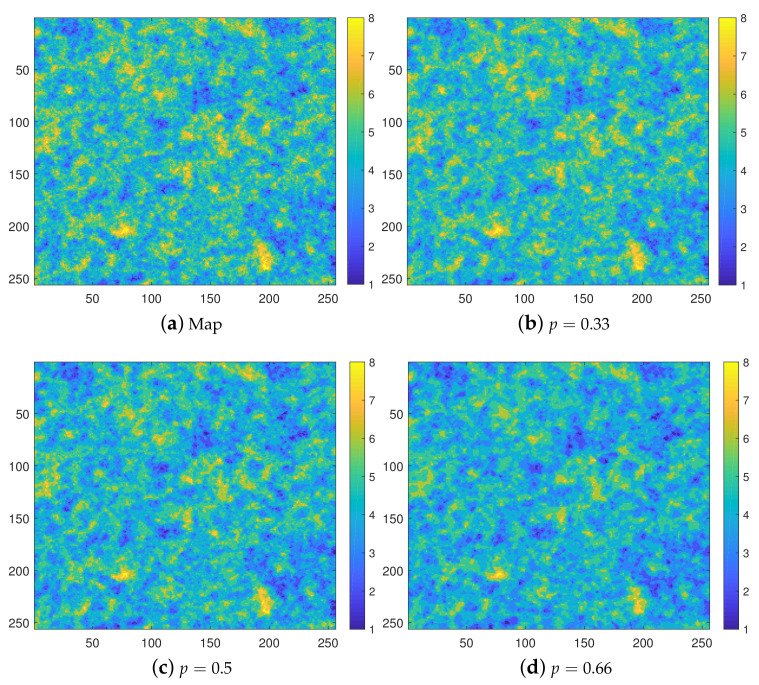
Eight-class isolevel map of the complete synthetic data for the selected size L=256 (**a**) and the reconstructions based on the thinned data with p=0.33 (**b**), 0.5 (**c**), and 0.66 (**d**).

**Figure 9:**
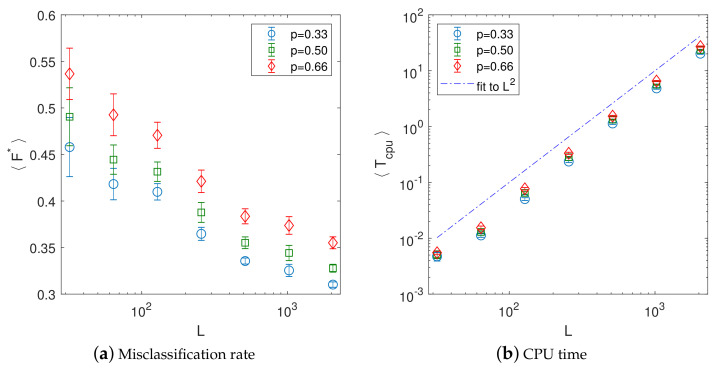
(**a**) Mean misclassification rate 〈F〉 and (**b**) mean computational time $\langle T_{cpu}\rangle$ of the INNC method versus data size for $N_{c}=8$ classes. The mean values and error bars are obtained from 100 realizations. The dash-dot line in (**b**) is a visual aid for linear dependence.

Finally, we study the performance of the INNC method on increasing grid sizes $N_{G}=L\times L$, with $L=2^{n}$ and n=5,⋯,11. We use the above described spatially correlated synthetic data discretized to obtain $N_{c}=8$ levels. For illustration, in Figure 8 we show the original data set for the selected size L=256 after discretization along with the reconstructions for the thinning values p=0.33, 0.5, and 0.66. The results showing both the interpolation and computational performance for different values of *L* and *p* are presented in Figure 9. In particular, Figure 9a shows that the misclassification rates gradually decrease with increasing grid size from initial values of $\langle F^{*}\rangle =(0.46\pm 0.03,0.49\pm 0.03,0.54\pm 0.03$ for p=(0.33,0.5,0.66) and L=32 down to $\langle F^{*}\rangle =(0.31\pm 0.003,0.33\pm 0.004,0.36\pm 0.006$ for p=(0.33,0.5,0.66) and L=2048. The decrease is less steep at larger *L*, but nevertheless it continues for all sizes up to L=2048.

Figure 9b shows the behavior of the corresponding CPU time versus grid size on a log–log plot. The plots indicate an almost linear increase with the grid size $N_{G}=L^{2}$ (the actual fits produce slightly superlinear scaling with the exponent approximately equal to 1.05). As already observed in the previous cases, the CPU time also slightly increases with *p*.

## 8. Conclusions

We investigated the INNC interpolation method which can be used to fill gaps in gridded spatial data. The latter can represent either processes that take discrete class labels or real values, discrete, or continuous. We showed that INNC is suitable for automatic mapping of large spatial data sets and demonstrated that its interpolation on different real-world and synthetic data sets is competitive against standard methods.

The INNC method is inspired from the Ising model. It is based on minimizing a cost function that measures the distance between sample-based, normalized, discrete correlation energies, and the respective energies of the entire domain (grid). INNC is implemented by using greedy Monte Carlo simulation conditioned by the sample values. Owing to a thoughtful initialization of the unknown values on the prediction subgrid, a greedy optimization approach, and vectorization, INNC is computationally fast. The time needed for the Monte Carlo relaxation is very short, and the resulting CPU time varies almost linearly with respect to both the number of classes and the grid size. Furthermore, the INNC method is universal with regard to the data probability distributions (i.e., it makes no assumptions thanks to its non-parametric nature). In addition, it is almost automatic and can be applied with no ad hoc inputs. The only parametrization in the proposed approach involves the number of discretization classes to be used for continuous data. The number of classes can be set arbitrarily large if high resolution is needed.

The model is demonstrated herein for regular grids. However, the extension to irregularly spaced data is straightforward. The interaction constant $J_{i,j}$ in Equation (1) can be defined via a kernel function (such as the radial basis function). The interaction neighborhood (nearest neighbors) of any point $\vec{r}$ can be defined to include those points for which their Voronoi cells share a boundary with $\vec{r}$. Furthermore, possible extensions could include the incorporation of further-neighbor or/and “multi-spin” correlation energy in the Hamiltonian. Overall, based on the studies presented herein, INNC has great potential as a method for gap filling in remote-sensing data products, with minimal if any intervention by the user. We will investigate this further in forthcoming publications.

## Figures and Tables

**Table 1:** Classification performance measures for the soil quality data using INNC and FKNN methods: mean misclassification rate $\langle F^{*}\rangle$, misclassification rate standard deviation ${\mathrm{STD}}_{F^{*}}$, and CPU time $\langle T_{cpu}\rangle$. Additional measures for INNC: mean number of Monte Carlo steps $\langle N_{MC}\rangle$ and mean value of cost function at termination $\langle U^{*}\rangle$. The averaging is performed over 100 realizations.

	p=0.33		p=0.5		p=0.66	
	INNC	FKNN	INNC	FKNN	INNC	FKNN
7 classes						
$\langle F^{*}\rangle$ (%)	24.94	45.78	26.43	47.12	28.64	49.19
${\mathrm{STD}}_{F^{*}}$	0.73	0.66	0.49	0.50	0.49	0.47
$\langle T_{cpu}\rangle$ (s)	0.0241	1.88	0.0254	2.34	0.0296	2.36
$\langle N_{MC}\rangle$	5.74	−	6.23	−	6.68	−
$\langle U^{*}\rangle$	$1.4\times 10^{-4}$	−	$3.9\times 10^{-4}$	−	$6.9\times 10^{-4}$	−

**Table 2:** Classification performance measures for the surface elevation data based on the INNC and FKNN methods: mean misclassification rate $\langle F^{*}\rangle$, misclassification rate standard deviation ${\mathrm{STD}}_{F^{*}}$, and CPU time $\langle T_{cpu}\rangle$. Additional measures for INNC: mean number of Monte Carlo steps $\langle N_{MC}\rangle$ and mean value of cost function at termination $\langle U^{*}\rangle$. The averaging is performed over 100 realizations.

	p=0.33		p=0.5		p=0.66	
	INNC	FKNN	INNC	FKNN	INNC	FKNN
8 classes						
$\langle F^{*}\rangle$ (%)	5.84	7.91	6.38	8.46	7.22	9.21
${\mathrm{STD}}_{F^{*}}$	0.13	0.15	0.11	0.10	0.15	0.10
$\langle T_{cpu}\rangle$ (s)	0.23	51.77	0.26	60.66	0.29	55.98
$\langle N_{MC}\rangle$	7.21	−	8.38	−	11.05	−
$\langle U^{*}\rangle$	$2.3\times 10^{-5}$	−	$3.1\times 10^{-6}$	−	$8.8\times 10^{-6}$	−
15 classes						
$\langle F^{*}\rangle$ (%)	11.55	15.16	12.51	15.98	13.93	17.17
${\mathrm{STD}}_{F^{*}}$	0.19	0.19	0.16	0.14	0.19	0.12
$\langle T_{cpu}\rangle$ (s)	0.44	52.35	0.49	62.34	0.55	56.74
$\langle N_{MC}\rangle$	13.78	−	15.76	−	20.63	−
$\langle U^{*}\rangle$	$5.2\times 10^{-5}$	−	$9.0\times 10^{-5}$	−	$1.1\times 10^{-4}$	−

## Data Availability

The soil quality data set (sq1.asc file) is available from the Harmonized World Soil Database at https://webarchive.iiasa.ac.at/Research/LUC/External-World-soil-database/HTML/SoilQualityData.html?sb=11 (accessed on 2 July 2021). The surface elevation data set is available from Global Digital Elevation Models at https://ngdc.noaa.gov/mgg/global/global.html (accessed on 18 June 2021). The code is available from Matlab File Exchange (https://www.mathworks.com/matlabcentral/fileexchange/99749-innc-interpolation-method (accessed on 23 September 2021)).

## Abbreviations

The following abbreviations are used in this manuscript:

INNC	Ising nearest neighbor correlation;
FKNN	Fuzzy k-nearest neighbor;
IDW	Inverse distance weighted;
RMSE	Root mean square error.
